# Effect of stereo‐EEG versus subdural EEG on functional and seizure outcome in pediatric and adult epilepsy surgery: A 21‐year single‐center experience

**DOI:** 10.1002/epd2.70025

**Published:** 2025-05-13

**Authors:** Ahmed Gaballa, Christian G. Bien, Philip Grewe, Anne Hagemann, Thilo Kalbhenn, Tilman Polster, Friedrich G. Woermann, Thomas Cloppenborg

**Affiliations:** ^1^ Department of Epileptology, Krankenhaus Mara, Bethel Epilepsy Centre, Medical School OWL Bielefeld University Bielefeld Germany; ^2^ Clinical Neuropsychology, Medical School OWL Bielefeld University Bielefeld Germany; ^3^ Society for Epilepsy Research Bielefeld Germany; ^4^ Department of Neurosurgery (Evangelisches Klinikum Bethel), Medical School OWL Bielefeld University Bielefeld Germany

**Keywords:** epilepsy surgery, intracranial EEG, SEEG, subdural EEG

## Abstract

**Objective:**

(1) To compare use, benefits, and complications of stereo‐EEG (SEEG) and subdural EEG (SD) in presurgical epilepsy candidates. (2) To evaluate the effectiveness of both methods in delineation of the epileptogenic zone (EZ) and guiding tailored resective surgery.

**Methods:**

We included patients with SEEG or SD evaluations in children and adults at the Bethel Epilepsy Centre, Germany, between 2000 and 2020. We retrospectively assessed epilepsy related parameters, main indication of iEEG (intracranial EEG, for identification of the EZ and functional mapping), complications, seizure, and functional outcome. The second objective was addressed by a subgroup analysis in patients with focal cortical dysplasia (FCD) and mild malformation of cortical development with oligodendroglial hyperplasia (MOGHE) for delineation of the EZ.

**Results:**

Two hundred and nineteen iEEG explorations in 215 patients (age range: 1.3–64.6 years) were assessed (SEEG, *n* = 78; SD, *n* = 141). We observed a change in iEEG usage away from SD towards SEEG. In the SEEG group, the decision rate against surgery was higher (SEEG: 23.1% vs. SE: 3.5%, *p* < .001) and more patients achieved seizure freedom 2 years after surgery (SEEG: 67.8% vs. SD: 46.5%, *p* = .008). For all iEEG, complications were rare; symptomatic intracranial bleeding only occurred in the SD group (*n* = 6, 4.3%). In patients with FCD/MOGHE for delineation of the EZ, all patients were operated with favorable seizure freedom after SEEG, but with acceptance of planned neurological deficits (SEEG *n* = 8, SD *n* = 20, seizure freedom rate SEEG 87.5% vs. SD 30%, *p* = .011; neurological deficits: SEEG *n* = 3 planned motor deficits vs. SD *n* = 1 unplanned motor deficit due to ischemia).

**Significance:**

SEEG has largely replaced SD in iEEG for the identification of the EZ. It is better tolerated and allows more freedom of choice with respect to resective surgery, resulting in the better identification of surgical candidates and delineation of the EZ with favorable seizure outcomes.

**Key points**

- There is a clear trend towards the usage of stereo‐EEG (SEEG) in the last 10 years.
- Functional mapping remains the main indication of subdural EEG in our center with good functional outcome after surgery.
- SEEG can successfully select patients for epilepsy surgery to achieve a good postoperative seizure outcome.
- Symptomatic intracranial hemorrhage occurred rarely, but only after subdural implantations.
- SEEG showed a better delineation of the EZ and higher rates of seizure freedom in patients with FCD and MOGHE.

## 1 INTRODUCTION

Epilepsy is one of the most common chronic neurological diseases, with a lifetime prevalence of 6.38 per 1000 persons.^1^ Resistance to pharmacotherapy, defined as a lack of therapeutic response to at least two adequately selected drugs in sufficient dosage, occurs in approximately one‐third of epilepsy patients and should lead to presurgical evaluation.^2^ In challenging cases, intracranial EEG (iEEG) can provide missing information in the presurgical workup.^3^ This information may have a direct impact on the seizure and functional outcome after epilepsy surgery. Subdural EEG (SD) and stereotactically‐implanted depth electrodes (SEEG) are the most frequently used and accepted methods in iEEG diagnostics in practice.^4^

The objective of iEEG is to define the epileptogenic zone (EZ) and brain eloquent areas with the help of intracranial electrical recordings and electrical stimulation (ESM).^5^ This diagnostic process aids in presurgical decision‐making, helping to determine not only whether to operate or not, but also to tailor individual resections. The goal after iEEG is to select patients for surgery, achieve seizure freedom, and avoid neurological functional loss.^6^

SD and SEEG are two different modalities used to achieve this goal. SD requires a craniotomy for implantation and explantation, can cover a greater cortical area and is suitable for functional mapping.^7^ On the other hand, SEEG only needs burr holes for implantation, offers coverage of neocortical structures in addition to deep brain structures and allows functional mapping.^8^, ^9^

Since SEEG and SD may provide the required information for surgical decision‐making, both modalities have been widely adopted, with different preferences in most centers.^10^ This makes objective and unbiased comparisons difficult. Nevertheless, these preferences may change over time. For example, multiple studies observed a shift in adoption towards SEEG in North America and China.^11^, ^12^, ^13^ Accessibility of both methods may differ from one center to the other. The heterogeneity of the surgical candidates and the different indications of iEEG in various clinical scenarios complicate the comparison of both modalities.

At our center, SEEG and SD have been accessible and frequently used for several years in presurgical diagnostics in pediatric and adult patients, allowing a monocentric study comparing both methods. Our study aims at confirming the paradigm shift towards SEEG at our center, as a general trend in recent literature. Additionally, we seek to compare both methods regarding the efficacy (based on preoperative detection of the epileptogenic zone/functional area), postoperative seizure freedom rates, complications, and functional outcomes in cases where functional mapping was performed. Due to our center's lesion‐oriented approach, we were particularly interested in comparing SD and SEEG in a subgroup of patients with focal cortical dysplasia, where iEEG was performed for delineation of the EZ and guiding tailored resective surgery.

## 2 METHODS

### 2.1 Study cohort and data selection

We conducted a single‐center retrospective cohort study of all pediatric and adult patients who underwent iEEG at the Bethel Epilepsy Centre, Germany, between 2000 and 2020 with either SD or SEEG. For the period 2021–2023, we only assessed the frequency of iEEG and resulting surgeries due to the lack of a 2‐year follow‐up at the time of data collection.

All patients had pharmacoresistant focal epilepsy and were selected for iEEG in an interdisciplinary case conference based on the results of their routine presurgical workup. This includes structural MRI and scalp EEG monitoring for all patients and additional investigations such as functional MRI, neuropsychological assessment, FDG‐PET, and Wada test, depending on patients' age and individual indication.

We identified the patients from our center's constantly updated epilepsy surgery database and extracted the following parameters from the database as well as from medical records: sex, age at epilepsy onset, age and epilepsy duration at the time of iEEG, duration of intracranial registration, and number of implanted electrodes/contacts. In the case of multiple intracranial recordings, these were analyzed as separate cases.

The potential epileptic lesions on MRI were classified with regard to localization (frontal, temporal, parietal, and multilobar). Cases with normal MRI were categorized as non‐lesional. Etiologies based on histopathological results after resective surgery were classified into hippocampal sclerosis, focal cortical dysplasia's (including MOGHE), tumors, gliosis, vascular malformations, tuberous sclerosis, and non‐specific, as described elsewhere.^14^

iEEG of any modality may contribute to (A) identification of the EZ and (B) functional mapping of the cortical surface. (A) Requires recording of seizures and is done in the large majority of patients, (B) is done via electrical stimulation. Frequently, both modalities are used.^5^ The results were determined as “reliable” or “not reliable” by the presurgical case conference.

Functional mapping aims at identifying potentially essential primary sensorimotor and language‐related areas (Broca's and Wernicke's areas, temporo‐basal language areas and the visual word form area, an essential region for whole‐word recognition and reading).^15^, ^16^

In SD cases, we performed a bipolar stimulation with a pulse width of 300–500 μs, either high frequency stimulation (50 Hz, 5–10 s) or low frequency stimulation (1 Hz, up to 30 s), starting with a 1 mA current and a gradual increase up to 15 mA if necessary. We considered electrodes with a functional response after bipolar stimulation between two different adjacent electrodes as eloquent. Sites with regional/diffuse after‐discharges were excluded.

With SEEG, we used pulse widths of 300–500 μs and a maximum current of 6 mA. For stimulation currents exceeding 3 mA, a maximum pulse width of 300 μs was applied. This resulted in charge densities <40 μC/cm^2^/phase in all situations, which is considered safe for short term stimulation settings, especially with biphasic current.^5^

Epilepsy surgeries were classified as follows: unilobar resection, apical temporal lobe resection (resection of the temporal pole and the amygdala, sparing the hippocampus), standard anteromedial temporal lobe resection, (sub)lobar resection, multilobar resection, posterior disconnection, and thermal coagulation.^17^ If corpus callosotomy was suggested after iEEG, the patients were not suitable for curative resective/ablative therapy and, therefore, classified as nonsurgical candidates.

We assessed the seizure outcome according to the Engel classification 2 years after surgery.^18^ Only patients with Engel 1A outcome were considered seizure‐free. Functional outcome was assessed for patients who underwent functional mapping by clinical neurological assessment (history and clinical neurological examination) after the same follow‐up period. Permanent deficits were defined as subjective symptoms reported by patients, with impact on daily life activities or objective clinical signs in the neurological examination.

In certain clinical scenarios, the planned surgical resection may involve eloquent areas according to mapping results. Consequently, the likelihood of corresponding functional deficits after surgery is high. In these instances, postoperative functional deficits were considered “expected deficits.” Conversely, if postoperative functional deficits manifested in cases where no eloquent areas were involved in the resection according to mapping results, these were considered “unexpected deficits.” Surgical complications unrelated to the site of iEEG were not assessed in this study.

Complications related to iEEG were retrospectively extracted from medical records and categorized into cerebral infection, intracranial hemorrhage, brain edema, psychosis, and acute neurological deficit. SD implantations regularly lead to asymptomatic and minor findings on postoperative imaging, such as pneumocephalus and smaller subdural or epidural hematomas, which do not require surgical intervention.^19^ These were not assessed as complications.

### 2.2 Implantation/Explantation of iEEG

The placement of the subdural electrodes was performed using standard techniques via craniotomy with an open surgical approach.^20^ Depth electrodes for SEEG (Spencer Probe Depth Electrodes, AD‐TECH) were implanted using robot guidance (Renishaw robot system). All implantations were performed under general anesthesia. For further details on depth electrode placement, see Ref. 21.

### 2.3 Statistical analyses

The categorical variables were summarized using absolute and relative frequencies and were compared using Fisher's exact test. The continuous variables were summarized using mean ± standard deviation and median (range) and were compared using the Mann–Whitney *U*‐test. All statistical tests were two‐tailed and the statistical significance level was set as α = .05. The statistical analyses were performed using IBM SPSS Statistics v.25.

### 2.4 Ethics

The responsible institutional review board for human research (Ethics Committee of the University of Münster, no. 2022‐839‐f‐S) approved the study.

## 3 RESULTS

### 3.1 Patient characteristics and trends over time

Two hundred and fifteen children and adults underwent 219 iEEG implantations between 2000 and 2020 (107 females, 48.9% and 112 males, 51.1%). In additional exceptional cases, we performed simultaneous SEEG and SD evaluations (*n* = 7); those cases were excluded from this analysis.

Table 1 lists the demographic data and gives information about the localization of the putative epileptogenic lesion on MRI. There were no differences between SEEG and SD patients with regard to sex‐, age‐, and time‐related variables such as age at epilepsy onset and disease duration at the time of iEEG. The distribution of MRI abnormalities and the frequency of MRI‐negative cases were comparable in both groups (Table 1, MRI‐negative: SEEG *n* = 11, 14.1% and SD *n* = 16, 11.3%). We performed only two implantations of SEEG in subependymal heterotopias, but no implantations for hypothalamic hamartomas or other deep brain structures.

**TABLE 1:** Demographic and clinical data.

	Stereo‐EEG (*n* = 78)	Subdural EEG (*n* = 141)	*p*‐value
Sex, *n* (%)			
Female	34 (43.6%)	73 (51.8%)	.262^a^
Male	44 (56.4%)	68 (48.2%)	
Age of seizure onset years, M ± S	9.1 ± 10.6	9.9 ± 9.7	.247^b^
Median (Range)	5.5 (.1–51.0)	7.0 (.1–50.0)	
n.a.	*n* = 0	n = 2	
Duration of Epilepsy years, M ± S	15.9 ± 12.2	15.3 ± 13.4	.284^b^
Median (Range)	12.8 (1.3–51.2)	9.8 (.4–54.1)	
n.a.	*n* = 0	*n* = 2	
Age of iEEG implantation years, M ± S	25.0 ± 15.5	25.3 ± 15.2	.803^b^
Median (Range)	24.1 (3.6–64.6)	24.5 (1.3–63.3)	
iEEG, duration in days, M ± S	7.4 ± 2.9	6.5 ± 1.9	.025^b^
Median (Range)	7.0 (2–14)	6.0 (2–21)	
n.a.	*n* = 0	*n* = 1	
Electrode contacts, *n*, M ± S	59.0 ± 20.1	52.2 ± (18.8)	.008^b^
Median (Range)	60.0 (20–103)	50.0 (16–124)	
n.a.	*n* = 0	*n* = 1	
MRI			
Non‐lesional epilepsy	11 (14.1%)	16 (11.3%)	.668^a^
Lesional epilepsy, *n* (%) all	67 (85.9%)	125 (88.7%)	
Frontal, % of lesional	23 (29.5%)	40 (28.4%)	
Temporal	25 (32.1%)	40 (28.4%)	
Parietal	0 (.0%)	4 (2.8%)	
Multilobar	19 (24.5%)	41 (29.1%)	
Indication of iEEG, *n* (%)			
Only epileptogenic zone	72 (92.3%)	49 (34.8%)	<.001^a^
Only mapping	0 (0%)	34 (24.1%)	
Both	6 (7.7%)	58 (41.1%)	
Epilepsy surgery, *n* (%)	60 (76.9%)	136 (96.5%)	<.001^a^
Extended unilobar resection	18 (30%)	62 (45.6%)	.131^a^
Apical TL‐resection	1 (1.7%)	4 (2.9%)	
Anterio‐medial TL‐resection	20 (33.3%)	39 (28.7%)	
(Sub)total lobectomy	4 (6.7%)	5 (3.7%)	
Multilobar resection	13 (21.7%)	24 (17.6%)	
Posterior disconnection	2 (3.3%)	2 (1.5%)	
Thermocoagulation	2 (3.3%)	0 (0%)	
Histopathology			
Surgically treated patients, *n*	60	136	.443^a^
No histopathology done, *n*	2	0	
Dual histopathological findings, *n*	8	19	
All histopathological findings, *n*	66	155	
Hippocampal sclerosis *n* (%)	19 (28.8%)	26 (16.8%)	
FCD/MOGHE	24 (36.4%)	56 (36.1%)	
Tumors	5 (7.6%)	20 (12.9%)	
Gliosis	5 (7.6%)	10 (6.5%)	
Vascular malformation	1 (1.5%)	7 (4.5%)	
Tuberous sclerosis	2 (3.0%)	8 (5.2%)	
Non‐specific	10 (15.1%)	28 (18.1%)	

Abbreviations: FCD, focal cortical dysplasia (SEEG: FCDI *n* = 1, MOGHE *n* = 3, FCD II *n* = 18, FCD III *n* = 2), (SD: FCDI *n* = 10, MOGHE *n* = 5, FCD II *n* = 35, FCD III *n* = 6); iEEG, intracranial EEG; M, mean; MOGHE, mild malformation of cortical development with oligodendroglial hyperplasia; n.a., data not available; S, standard deviation; TL, temporal lobe.

^a^ Fisher's exact test.

^b^ Mann–Whitney *U*‐Test.

Malformations of cortical development were the most frequent etiology in the operated cohort with comparable proportions in both SD and SEEG (Table 1, about 1/3 of patients in each group).

**FIGURE 1:**
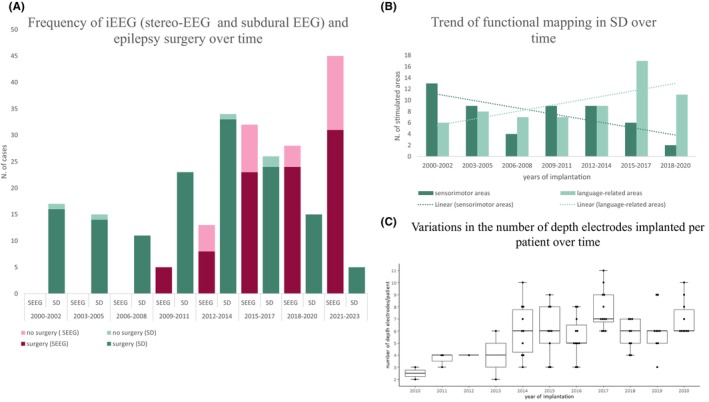
Trends over time. The figure shows trends over time for SD and SEEG implantations and subsequent resective surgeries, domains of functional mapping in SD patients, and the variations in the number of implanted depth electrodes per patient over time. Dotted lines, trendlines; SD, subdural EEG; SEEG, stereo‐EEG.

Figure 1A compares the frequency of SEEG and SD implantations over time. At the beginning of the study period, the number of iEEG investigations initially increased threefold from 2000 to 2012 and has since remained stable at about 15 implantations per year. Meanwhile, SEEG has largely replaced SD explorations (*n* = 45 SEEG patients vs. *n* = 5 SD patients in 2021–2023).

The mean number of depth electrodes was 6.3 per patient. The median number increased from 2.5 in 2010 to 6.0 in 2020 (Figure 1C). In the SEEG group, the mean number of electrode contacts was higher (SEEG 59.0 ± 20.1 vs. SD 52.2 ± 18.8, *p* = .008; Table 1) and the mean duration of iEEG registration was significantly longer (SEEG 7.4 ± 2.9 days vs. SD 6.5 ± 1.9 days, *p* = .025).

### 3.2 Indications for iEEG, surgeries, and outcome

Identification of the EZ was the main indication for SEEG explorations, compared to functional mapping, which was performed mainly by SD (Table 1). In the period 2021–2023, five patients underwent SD exploration, of which only one patient was implanted to identify the EZ. Indications for functional mapping shifted from sensorimotor and language areas to mainly language‐related areas over time (Figure 1B).

**FIGURE 2:**
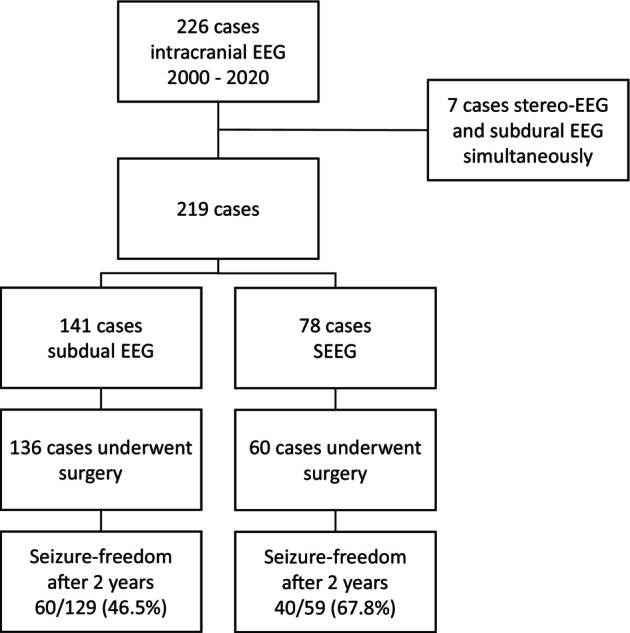
IEEG cases with subdural EEG and stereo‐EEG.

SEEG was better suited to provide reliable information on identifying the EZ than SD (SEEG 77/78, 98.7% vs. SD 93/107, 86.9%, *p* = .005). The rejection rate from surgery was significantly higher in the SEEG group (operated patients SEEG *n* = 60, 76.9% vs. SD *n* = 136, 96.5%, *p* < .001; Table 2) and was due to detection of large seizure networks, in which resective surgery was not possible. Following SEEG evaluations, we observed a trend towards more complex and extensive interventions (Table 1, higher rates of extended subtotal lobectomies, multilobar resections and posterior disconnections). Postoperative follow‐up 2 years after surgery was available for the vast majority of patients (SEEG *n* = 59, 98.3% vs. SD *n* = 129, 94.9%). Regarding the seizure freedom rate in all patients with iEEG, there was no statistically significant difference between the groups (SEEG 40/78, 51.3% vs. SD 60/141, 42.6%, *p* = .257). The overall seizure freedom rate was 53.2% of all operated patients (100 of 188 patients). Of all surgically treated patients with available outcomes 2 years after surgery, more patients were seizure free after SEEG evaluation compared to SD (SEEG 40/59, 67.8% vs. SD 60/129, 46.5%, *p* = .008). The rate of patients with unfavorable outcomes was also lower in the SEEG group (Engel class IV, SEEG 5.1% (*n* = 3/59) vs. SD 21.7% (*n* = 28/129) *p* = .005). The same results were shown, when comparing the years from 2012 to 2020, when SD and SEEG were in parallel use (SEEG *n* = 73, surgery *n* = 55; SD *n* = 75, surgery *n* = 72; seizure freedom rates: SEEG 69.1% (*n* = 38/55) vs. SD 47.2% (*n* = 34/72), *p* = .019) (Figure 2).

**TABLE 2:** Outcome and complications.

	Stereo‐EEG (*n* = 78)	Subdural EEG (*n* = 141)	*p*‐value^a^
Surgical outcome			
Epilepsy surgery all patients, *n*	60	136	<.001
Follow‐up 2 years, *n*	59	129	
Seizure outcome (Engel classification, *n* (% operated with 2 years follow‐up))			
Seizure free (IA)	40 (67.8%)	60 (46.5%)	.008
IB, IC, ID	4 (6.8%)	9 (7.0%)	–
II	7 (11.9%)	12 (9.3%)	–
III	5 (8.5%)	20 (15.5%)	–
IV	3 (5.1%)	28 (21.7%)	.005
iEEG related outcome			
iEEG for EZ identification, *n*	78	107	
Surgery, *n*	60	103	
Follow‐up 2 years, *n*	59	99	
Seizure persistence at follow‐up 2 years^b^			
All, *n*	19 (32.2%)	61 (61.6%)	<.001
Expected, *n*	2	13	
Unexpected, *n*	17 (28.9%)	48 (48.5%)	.019
iEEG for functional mapping, *n*	6	92	
Epilepsy surgery	6	89	
Functional deficit, after 6 months	2 (33.3%)	17 (19.1%)	.596
Follow‐up 2 years, *n*	6	85^c^	
Functional deficit all, after 2 years	2 (33.3%)	13 (15.3%)	.256
Expected functional deficit	2	7	
Functional deficit as a complication, *n*	0	2^d^	
(Unexpected) Functional deficit	0 (.0%)	4 (4.7%)	
Complications			
All iEEG	78	141	
Data available	78	140	
Patients with complications, *n* (%)	4 (5.1%)	9 (6.4%)	.776
Symptomatic intracranial hemorrhage	0 (.0%)	6 (4.3%)	.091
Neurosurgical intervention was needed	0 (.0%)	4 (2.9%)	.299

Abbreviations: ESM, electrical stimulation; iEEG, intracranial EEG.

^a^ Fisher's exact test.

^b^ Seizure persistence: not achieving IA, Engel.

^c^ One case with functional deficit after 6 months was not available for follow‐up at 2 years.

^d^ Two cases developed deficits as a result of postsurgical infarction (not related to iEEG).

With respect to the indication “identification of the EZ” in patients with available follow‐up, more patients showed persistent seizures with resective surgery after SD exploration compared to SEEG (SEEG 19/59, 32.2% vs. SD 61/99, 61.6%, *p* < .001). Adjusting these numbers by excluding patients for whom no curative approach was planned (SEEG *n* = 2, SD *n* = 13), the seizure persistence rate was still higher for SD patients (SEEG 17/59, 28.9% vs. SD 48/99, 48.5%, *p* = .019; Tables 2 and 3).

**TABLE 3:** Functional mapping by subdural EEG.

	Sensorimotor areas		Language areas			
	Motor	Sensory	Broca	Wernicke	VWFA	Temporo‐basal areas
Stimulated areas, *n*	49	3	20	35	6	4
Area defined, *n* (%)	47 (95.9)	3 (100)	14 (70)	30 (85.7)	3 (50)	3 (75)
Surgery, *n*	48	3	20	34	6	3
2‐years follow‐up, *n*	45	2	20	32	6	3
Expected deficits, *n* (%)	4 (8.8)	0 (0)	1 (5)	4 (12.5)	0 (0)	0 (0)
Unexpected deficits, *n* (%)	1 (2.2)	0 (0)	1 (5)	2 (6.3)	0 (0)	0 (0)

*Note*: Results of functional mapping in 92 cases in 117 functional areas, successful mapping in 100/117 areas (85.5%).

Abbreviations: *n*, number of patients; VWFA, visual word form area.

### 3.3 Subgroup analysis in patients with malformations of cortical development for delineation of the epileptogenic zone

**FIGURE 3:**
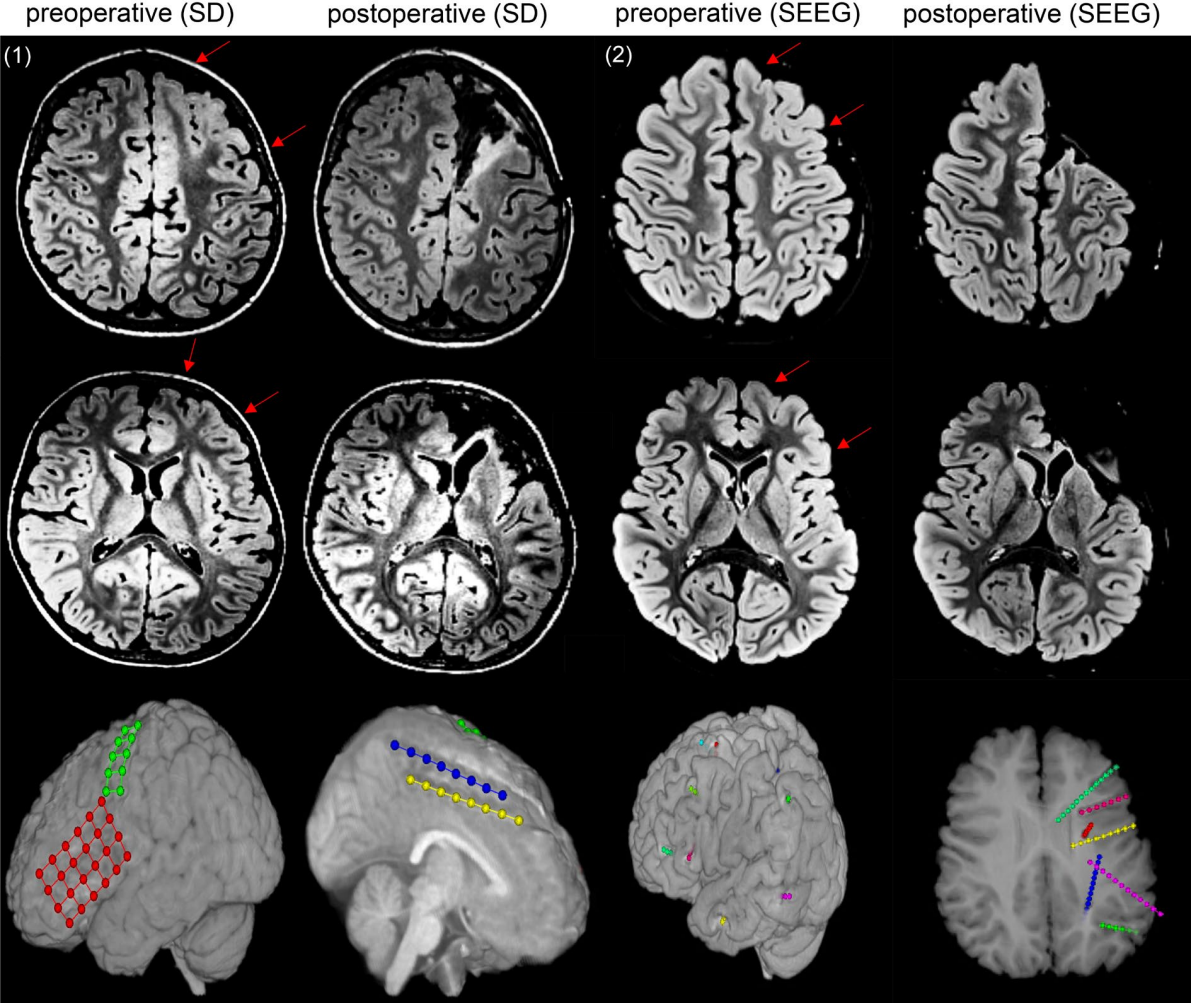
The delineation of the epileptogenic zone with SD and SEEG in two illustrative cases. Pre‐ and postoperative MRI and implantation schemes of two young children with epileptic spasms (and other seizure types) with extensive gray–white matter blurring in the left frontal lobe (arrows indicate the approximate a‐p extension) with poorly marked posterior borders. The surface EEG showed interictally multifocal sharp waves bilaterally, and the seizures produced left hemispheric diffuse ictal patterns. Patient 1 was implanted with one subdural grid (4 × 6 contacts) and three subdural strips (2 × 5, 1 × 8, 1 × 8) on the convexity and the medial surface of the left frontal lobe at the age of four to delineate the posterior extent of the epileptogenic zone. Ictal EEG showed widespread activity in the premotor frontal lobe. Patient 2 was implanted with nine depth electrodes in the left hemisphere (frontal 4, temporal 2, parietal 1, insular 2) to delineate the posterior extent of the epileptogenic zone. Ictal recordings showed activity in the left lateral frontal lobe as well as in the anterior insula. Both patients underwent subtotal frontal lobe resection, patient 2 a resection of the anterior insula in addition. Patient 2 had a supplementary motor area (SMA) syndrome including speech loss for a few weeks only. Both histopathologies were mild malformation of cortical development with oligodendrogial hyperplasia (MOGHE). Two years after surgery, patient 1 had an Engel 2A outcome, whereas patient 2 was Engel 1A.

A comparison of methods works best in defined subgroups for exclusion of confounding factors. For this reason, we chose patients with histopathology of FCD and MOGHE, where iEEG aimed at delineation of the EZ and tailoring resection. A subgroup of 8 SEEG patients and 20 SD patients fulfilled these criteria. The timespan of implantation overlapped and both methods were already established (SEEG 2014–2017, SD 2000–2018). Table 4 illustrates the localization of lesions, the implantation sites, details regarding epilepsy surgery, histopathology findings, and both seizure and functional outcomes. We performed epilepsy surgery in all cases. Notably, the seizure freedom rate was significantly higher in the SEEG group (87.5% for SEEG vs. 30% for SD, *p* = .011). This result indicates a better delineation of the epileptogenic zone by SEEG. Figure 3 shows two illustrative cases with similar localization and histopathology to emphasize conceptual differences between both modalities in our center.

**TABLE 4:** Subgroup analysis: extent of the EZ in focal cortical dysplasia and MOGHE.

	Stereo‐EEG (*n* = 8)	Subdural EEG (*n* = 20)
Lesion location, *n* (right/left)	(5/3)	(8/12)
Fronto‐mesial	1	2
Fronto‐lateral	2	4
Fronto‐basal	1	0
Precentral	1	3
Pericentral	1	3
Fronto‐temporal	0	2
Fronto‐temporo‐insular	0	1
Fronto‐parieto‐insular	0	1
Temporal	0	1
Temporo‐insular	1	0
Temporo‐occipital	1	3
Implantation location, *n* (%)		
Frontal	1 (12.5)	4 (20)
Frontal plus (parietal)	2 (25)	6 (30)
Frontal plus (temporal)	0 (0)	1 (5)
Multilobar	5 (62.5)	9 (45)
Mapping, *n*		
Motor	2	12
Sensory	1	2
Broca	0	4
Wernicke	0	1
Epilepsy surgery, *n* (%)	8 (100)	20 (100)
Unilobar extended resection	2 (25)	8 (40)
Subtotal lobectomy	1 (12.5)	3 (15)
Multilobar resection	4 (50)	9 (45)
Posterior disconnection	1 (12.5)	0 (0)
Histopathology, *n* (%)		
FCD I	0 (0)	7 (35)
FCD II	5 (62.5)	10 (50)
MOGHE	3 (37.5)	3 (15)
Seizure freedom, *n* (%)	7 (87.5)	6 (30)*
Expected seizure persistence due to planned incomplete resection, *n*	0 (0)	5 (25)
Postoperative neurological deficits, *n*		
Visual field	2 (25)	2 (10)
Motor	3 (37.5)	1 (5)

Abbreviations: EZ, epileptogenic zone; FCD, focal cortical dysplasia; MOGHE, mild malformation of cortical development with oligodendroglial hyperplasia.

* Fisher's exact test, *p* = .011.

In the SD group, we performed five planned incomplete resections between 2001 and 2003. Incomplete resection aimed for preserving motor or visual function in four of these patients. None of the patients with incomplete resection achieved seizure freedom.

We observed a higher rate of motor deficits and visual field defects after SEEG evaluation (motor deficits: SEEG *n* = 3, 37.5% vs. SD *n* = 1, 5%; visual field defects: SEEG *n* = 2, 25% vs. SD n = 2, 10%). These occurred from 2012 to 2017. All motor deficits in the SEEG group were anticipated preoperatively due to resections in eloquent areas, one case from the SD group developed a motor deficit due to ischemic complications.

Functional mapping was mainly performed with SD exploration (92 cases to define 117 functional brain areas in the SD group vs. six cases to define seven functional areas in the SEEG group; Table 3). SD could reliably identify the eloquent cortex in 85.5% of all indications (*n* = 100/117). Almost all patients went for resective surgery after SD mapping (*n* = 89 of 92 patients; 96.7%). Two years after surgery, corresponding functional deficits after SD mapping were reported in *n* = 13 (15.3%) of the patients. Among 13 patients, seven developed an expected functional deficit due to a resection of eloquent cortex or impact on the pyramidal tracts. In two other patients, functional deficits occurred after postsurgical infarction and were unrelated to the iEEG results (see Table 2). The number of unexpected permanent functional deficits after SD mapping was low (*n* = 4; 4.7%). Three patients suffered from mild aphasic symptoms (anomic aphasia), and one patient developed a mild left‐sided hemiparesis.

In the six cases with functional mapping after SEEG, no unexpected postsurgical functional deficits occurred. Resection was performed in two patients with high seizure‐ burden in the SEEG group, although the area to be resected overlapped with functional cortex. Consequently, one case developed a mild right sided hand paresis and the other one suffered from high grade left‐sided hemiparesis with relevant gait disturbance.

### 3.4 Complications of iEEG

Table 5 illustrates all iEEG complications, as well as the resulting management and deficits. The complication rate after iEEG implantation was generally low and showed no significant difference between SEEG and SD (SEEG, *n* = 4; 5.1% vs. SD, *n* = 9; 6.4%, *p* = .776). Intracranial bleeding was the most frequent complication in both groups (SD, *n* = 6; 4.3% vs. SEEG, *n* = 2; 2.6%). Five of the six patients with bleeds in the SD group showed acute neurological deficits and most of them recovered after management with fluid restriction and corticosteroids or explantation and evacuation of hemorrhage. Only one patient developed a permanent deficit after SD implantation; he suffered from acute hemiparesis due to subdural bleeding. The patient consented to neurosurgical decompression combined with resection of the EZ with the acceptance of permanent hemiparesis. The decision was justified, as the patient became seizure free after surgery. The two patients who developed intracerebral bleeding in the SEEG group did not show any neurological deficits.

**TABLE 5:** Cascade of complications and interventions after iEEG implantation.

iEEG	Subdural EEG									Stereo‐EEG			
Patients	P1	P2	P3	P4	P5	P6	P7	P8	P9	P1	P2	P3	P4
Intracranial bleeding	•	•	•	•	•	•				•	•		
Acute neurological deficit	•	•	•		•	•							•
Neurosurgical intervention	•	•	•	•									
Explantation	•	•	•	•			•					•	
Permanent neurological deficit	•												
Brain oedema							•	•					
Postictal psychosis									•			•	

Two patients (one patient in each group) developed postictal psychoses during iEEG registration; one SEEG patient suffered from delusional thoughts and visual hallucinations, was aggressive and tore off one depth electrode. We interrupted iEEG and explanted the remaining electrodes. One SD patient developed auditory hallucinations and irritability, responding well to neuroleptics without early interruption of iEEG.

## 4 DISCUSSION

Our study identified three trends in the application of iEEG: (a) the shift in iEEG methods away from SD towards SEEG, (b) the higher number of implanted depth electrodes per patient, and (c) a decrease in functional mapping, particularly of motor areas.

The ability to examine deep brain structures, the greater availability of robot‐assisted implantation devices, and the lower complication rates of SEEG combined with its better tolerability are possible explanations for the paradigm shift from SD towards SEEG.^4^

The increasing number of SEEG possibly reflects a learning curve in SEEG application at our center. While SEEG was initially used for confirmation of an EZ in MRI lesional cases, SEEG was later used for the identification and delineation of the EZ in patients with MRI lesions of unknown extent and MRI‐negative cases. The number of MRI‐negative cases at our center (SEEG *n* = 11, 14.1% and SD *n* = 16, 11.3%) is low in comparison to other published series (Tandon 2019, Joswig 2020 and Kim 2021: SEEG 27.7%–56.2% and SD 28.8%–39.7%) and the average number of implanted electrodes was higher in these studies (mean 8.5–15).^22^, ^23^, ^24^ These data reflect the mainly lesion‐oriented approach at our center.

Kim et al.^24^ reported higher rates for the identification of the EZ in slight favor of SEEG (SEEG, *n* = 47; 91.5% vs. SD, *n* = 19; 88.2%). A similar trend was also reported by Yang et al.^25^ (SEEG, *n* = 48; 97.9% vs. SD, *n* = 52; 92.3%). Our data are consistent with earlier studies supporting the significant superiority of SEEG in the identification of the EZ compared to SD.

At our center, SEEG resulted in higher rates of rejection from surgery, resulting in higher rates of seizure freedom. The same was true in the subgroup analysis of FCD/MOGHE patients for delineation of the EZ. Compared to SD evaluations, this observation refers to the more successful selection of patients after SEEG.

Conceptual differences in the use of SEEG and SD could have an impact on seizure freedom after surgery. Whereas SD involves the grid‐like coverage of large brain areas and precise mapping of the seizure onset zone by interictal and ictal EEG is attempted to proof or disprove one surgical strategy, SEEG has the potential to prove various surgical concepts. The two illustrative cases (Figure 3) are examples for different surgical decision‐making options in the two methods: the SD case attempts to determine the extension of a frontal lobe resection without motor deficit, while the SEEG case examines different surgical options as frontal, fronto‐insular, or multilobar resection including the frontal, insular, and temporal regions. These various surgical options have been considered in SEEG planning. Despite lower spatial resolution, SEEG proves the involvement of a region in the epileptogenic network. The seemingly higher precision of SD that is thought to result in tailored resection carries a risk of incomplete removal of the EZ, whereas the extent of resection after SEEG tends to be tailored by the deficit profile of a resection and other neurosurgical aspects. The resections are guided through the iEEG data from depth electrodes involved in the epileptogenic networks as well as “sentinel electrodes” outside the network. The different way of thinking in this context is the maximum resection without deficit after SEEG in comparison to a more restricted tailoring of the EZ after SD. This conceptual difference might lead to more extended resections and higher seizure freedom rates after SEEG. In the whole cohort as well as in the subgroup analysis the rate of unilobar resections was lower in the SEEG group; however, this difference did not reach statistical significance.

The formulated observation is supported by Tandon et al.,^22^ who compared rejection rates and postoperative seizure freedom in a large cohort of SEEG and SD patients (SEEG *n* = 121, SD *n* = 139). They also found a higher rejection rate from surgery after SEEG (25.6% vs. 8.6%, *p* < .001). A recent multicenter study reported higher odds for subsequent resective surgery after SD (SD *n* = 526, SEEG *n* = 942; OR 1.4, CI 95% [1.05–1.84]).^10^ Similar to our study, the seizure outcome in both studies was significantly better after SEEG (Engel I or II after 1 year, SEEG: 76.0% vs. SD: 54.6%) and (Engel I after >1 year, SEEG: 54.6% vs. SD: 41.1%), respectively.

Functional mapping is an important indication of iEEG and aims at preventing postsurgical functional deficits. Since the very beginning of iEEG with SD, the identification of functional cortical areas has been extensively studied.^26^, ^27^, ^28^ In the literature, not only SD is used for functional mapping, but recent publications have also reported successful functional mapping with SEEG.^29^ To date, the method has been evaluated only in a limited number of publications. In our study, no patient was explored with SEEG for functional mapping purposes only. Aungaroon et al.^30^ compared SD (*n* = 106) and SEEG (*n* = 67) for functional mapping, and reported a comparable efficacy in identifying language and motor regions, even reporting the superiority of SEEG for the identification of sensory areas compared to SD. We mainly used SD and achieved reliable information about the eloquent cortex in 85.5% of cases. Unexpected permanent deficits were rare (*n* = 4, 4.7%) and, except for one case, led to only mild impairment in daily life activities. The proportion of cases with ESM in the SD group was 65.2%, which reflects a higher proportion of lesions adjacent to eloquent areas. Although a complete resection was conducted in most cases, the proximity to eloquent areas does not always allow extended resections and might result in less favorable seizure freedom rates.

The subgroup analysis of FCD/MOGHE cases for delineation of the EZ and tailoring resections showed a higher rate of seizure freedom after SEEG, which refers to a better efficacy of this method with regard to this specific indication. Furthermore, the rate of neurological deficits was higher in the SEEG group after resection (Table 4). This may reflect a shift in preoperative counseling, shared decision‐making, and surgical strategies, while patients accepted the anticipated permanent neurological deficits in pursuit of seizure freedom.

We have observed a decrease in the intracranial functional mapping of motor areas over time, which has been replaced by other extraoperative and, if necessary, intraoperative techniques. Today, preoperative transcranial magnetic stimulation (TMS)‐data can be merged with MRI‐neuronavigation data, including diffusion tensor imaging (DTI) with fiber tractography, and used for preoperative resection planning. Intraoperatively, phase reversal, cortical motor evoked potentials (MEP) as well as subcortical evoked potentials are standard tools to effectively monitor and protect the motor system during tissue resection.

We observed only one permanent motor deficit after SD implantation by subdural bleeding. Symptomatic bleedings did not occur after SEEG implantations and occurred in six cases after SD implantations. This represents a higher risk of bleeding after SD implantations, which was not statistically significant, most likely due to the low number of the patients with complications. We did not see any neurological infections in either subgroup. A systematic review and meta‐analysis reported infections, hemorrhage, and intracranial pressure as the commonest risks after subdural grid implantation.^31^ In a prospective population‐based study,^32^ the complication rate was the highest after subdural grids (7.4%) within all other iEEG modalities (SEEG, subdural strips, foramen ovale, and epidural electrodes). Consistent with our results, in a recently published retrospective study,^19^ hemorrhage was the most frequent complication after SD implantation, with a low rate of clinical relevance. This observation reflects the high tolerability of both methods.

### 4.1 Limitations

The present study has certain limitations inherent to its single‐center retrospective design, thereby rendering it vulnerable to misclassification bias: the absence of standardized data collection of the complications and postoperative neurological deficits. Another limitation is that the implantation of SEEG was not available in our center before 2012. Because of the selection bias, uncontrolled confounding factors as well as time‐related changes in diagnostic and treatment protocols (quality of imaging, intraoperative mapping, surgical equipment and approaches), the results of the outcome data should be interpreted cautiously. Conducting randomized multicenter prospective studies for this purpose is fraught with difficulties due to the heterogeneity of indications and strategies adopted by different centers. A prospective study that focuses on cohorts with the same specific indication or etiologically homogenous cohorts could offer a feasible solution to this problem.

## 5 CONCLUSION

SEEG has largely replaced SD in iEEG for the identification and delineation of the EZ and borne a greater freedom of choice in favor of or against resective surgery. Furthermore, SEEG provides a wider range of presurgical evaluation considering different resection plans, resulting in better identification of surgical candidates with favorable seizure free outcome. Functional mapping, especially of language‐related cortical areas, remains the domain of SD evaluation.

SEEG showed a very good tolerability with no symptomatic bleedings in this group.

## CONFLICT OF INTEREST STATEMENT

None.

**Test yourself**

1. What is the most common complication of subdural electrodes?
  - Hemorrhage
  - Infection
  - Permanent neurological deficits
2. Which clinical case among the following is most suitable for subdural electrode implantation?
  - MR‐negative epilepsy patient who needs an extensive and bilateral implantation
  - A patient with a lesion adjacent to Wernicke's area
  - A patient with a lesion in deep brain structures
3. According to this paper, the SEEG group, in comparison to the subdural group, led to:
  - More epilepsy surgery, better seizure outcome
  - More epilepsy surgery, worse seizure outcome
  - Less epilepsy surgery, better seizure outcome
  - Less epilepsy surgery, worse seizure outcome

*Answers may be found in the* *supporting information.*

## Supporting information


File S1.

File S2.

Data S1.

## Data Availability

The data that supports the findings of this study are available in the Supporting Information of this article.
