# Supplementary material for: Effect of stereo‐EEG versus subdural EEG on functional and seizure outcome in pediatric and adult epilepsy surgery: A 21‐year single‐center experience
Source: Epileptic Disord. 2025 May 13;27(4):586–99. doi: 10.1002/epd2.70025 (PMC12398195; doi:10.1002/epd2.70025)
Supplement: Supplementary file 1 — File S1. [file EPD2-27-586-s002.docx]

**Supplementary file with patient level data for depth electrodes (DE):**

r: right, l: left, SOZ: seizure onset zone, HS: hippocampus sclerosis, FCD: focal cortical dysplasia, MOGHE: mild malformation of cortical development with oligondendroglial hyperplasia, mMCD: mild malformation of cortical development, n.a.: not available.

| Patient Nr. | DE vs SD | Epilepsy onset | Epilepsy duration | MR positive | SOZ localization | electrodes number right | electrodes number left | electrodes localisation | Histopathology | Surgery | Outcome Engel 2 years |
| --- | --- | --- | --- | --- | --- | --- | --- | --- | --- | --- | --- |
| 1 | DE | 4 | 11,13 | yes | frontal, r | 8 | 0 | frontal, insula | FCD IIa | yes | 1A |
| 2 | DE | 43 | 20,3 | yes | temporal, r | 3 | 3 | temporal, bilateral | HS 1 | yes | 2B |
| 3 | DE | 25 | 24,44 | yes | temporal, r | 6 | 0 | temporal, insula | HS 1 | yes | 1A |
| 4 | DE | 2 | 5,96 | yes | frontal, r | 9 | 0 | frontal | FCD IIb | yes | 1A |
| 5 | DE | 1 | 33,87 | yes | temporo-occipital, r | 6 | 0 | temporal, occipital | HS 1 | yes | 2B |
| 6 | DE | 9 | 43,69 | no | not localized | 6 | 1 | (frontal, temporal, parietal) r, frontal l | none | no | n.a. |
| 7 | DE | 12 | 12,99 | no | frontal, r | 5 | 5 | (frontal, parietal), bilateral | mMCD II | yes | 1A |
| 8 | DE | 21 | 1,93 | yes | temporal, r | 6 | 0 | temporal, parietal | Ganglioglioma (WHO Grade I) | yes | 1A |
| 9 | DE | 10 | 51,18 | yes | temporal, bilateral | 3 | 3 | temporal, bilateral | none | no | n.a. |
| 10 | DE | 51 | 13,63 | yes | temporal, l | 3 | 3 | temporal, bilateral | HS 1 | yes | 1A |
| 11 | DE | 23 | 10,03 | yes | temporal, r | 3 | 3 | temporal, bilateral | HS 3 | yes | 2B |
| 12 | DE | 7 | 20,69 | no | fronto-temporal, r | 9 | 0 | temporal, insula, frontal, parietal, occipital | mMCD II | yes | 3A |
| 13 | DE | 8 | 29,79 | yes | temporal, r | 5 | 0 | temporal, parietal, frontal | HS 1 + FCD IIIa | yes | 1A |
| 14 | DE | 2 | 12,7 | yes | hemispheric, r | 6 | 0 | temporal, insula, frontal, parietal, occipital | none | no | n.a. |
| 15 | DE | 17 | 4,39 | yes | temporal, l | 2 | 4 | temporal, bilateral | Gliosis | yes | 1A |
| 16 | DE | 1 | 24,8 | yes | temporal, l | 0 | 3 | temporal | mMCD II | yes | 1A |
| 17 | DE | 0,25 | 3,31 | yes | fronto-parietal, r | 9 | 0 | frontal, parietal | FCD IIa | yes | 1A |
| 18 | DE | 1 | 7,28 | yes | temporo-occipital, r | 5 | 0 | temporal, frontal, occipital | FCD IIa | yes | 1A |
| 19 | DE | 5 | 22,98 | yes | temporal, bilateral | 3 | 3 | temporal, bilateral | none | no | n.a. |
| 20 | DE | 16 | 3,26 | yes | temporal, l | 0 | 6 | temporal, parietal, frontal | mMCD II + Gliosis | yes | 1B |
| 21 | DE | 10 | 5,02 | yes | temporo-insular, r | 5 | 0 | temporal, frontal, insula | HS 1 + Gliosis in the amygdala | yes | 3A |
| 22 | DE | 3 | 4,23 | yes | frontal, r | 7 | 0 | frontal, parietal | FCD IIb | yes | 1A |
| 23 | DE | 1 | 48,58 | yes | temporal, l | 3 | 4 | (temporal, occipital), bilateral | HS 1 + Ganglioglioma | yes | 1A |
| 24 | DE | 1 | 10,65 | yes | frontal and temporo-parietal, r | 4 | 3 | (frontal, temporal, parietal) r, (frontal, occipital), l | Cortical tuber | yes | 1A |
| 25 | DE | 6 | 19,15 | yes | fronto-insular, r | 5 | 1 | (frontal, insula) r, frontal l | FCD IIb | yes | 1A |
| 26 | DE | 0,25 | 11,36 | yes | fronto-parietal, l | 1 | 4 | (frontal, parietal) l, frontal r | Cortical tuber | yes | 3A |
| 27 | DE | 18 | 9,73 | yes | frontal, l | 1 | 3 | frontal, bilateral | FCD IIb | yes | 2A |
| 28 | DE | 49 | 9,78 | yes | frontal, l | 1 | 3 | frontal, bilateral | FCD IIb | yes | 1A |
| 29 | DE | 3 | 38,53 | yes | diffuse, bilateral | 5 | 2 | (frontal, temporal), bilateral | none | no | n.a. |
| 30 | DE | 10 | 9,79 | yes | temporal, l | 0 | 7 | frontal, temporal | Ganglioglioma | yes | 1A |
| 31 | DE | 0,16 | 4,4 | yes | frontal, r | 11 | 0 | frontal, parietal | MOGHE | yes | 1A |
| 32 | DE | 10 | 8,36 | yes | frontal, r | 10 | 0 | frontal, parietal | FCD IIa | yes | 1A |
| 33 | DE | 13 | 16,15 | yes | temporal, l | 4 | 5 | (frontal, temporal), bilateral | HS 1 | yes | 1A |
| 34 | DE | 1 | 10,5 | yes | temporo-occipital, r | 8 | 0 | frontal, temporal, parietal, occipital | FCD IIb | yes | 1A |
| 35 | DE | 11 | 13,61 | no | temporal, l | 0 | 7 | temporal, insula | Gliosis + HS 2 | yes | 1A |
| 36 | DE | 0,25 | 5,73 | no | temporal, l | 0 | 9 | frontal, temporal | FCD IIa | yes | 4B |
| 37 | DE | 5 | 16,54 | yes | frontal, r | 5 | 1 | (frontal, temporal, insula) r, frontal l | FCD IIa | yes | 1A |
| 38 | DE | 7 | 19,69 | no | diffuse, bilateral | 4 | 2 | frontal, bilateral | none | no | n.a. |
| 39 | DE | 3 | 2,31 | yes | parietal, l | 0 | 7 | temporal, parietal, occipital | none | no | n.a. |
| 40 | DE | 3 | 8,96 | yes | frontal, r | 5 | 1 | frontal, bilateral | FCD IIa | yes | 1A |
| 41 | DE | 3 | 9,47 | yes | temporo-insular, r | 8 | 0 | temporal, frontal, insula | FCD IIb | yes | 1A |
| 42 | DE | 1 | 4,75 | yes | temporo-parieto-occipital l and temporal r | 2 | 6 | (temporal, parietal, occipital) l, temporal r | Ganglioglioma + Cavernous hemangioma | yes | 1A |
| 43 | DE | 1 | 42,71 | yes | temporal, bilateral | 3 | 3 | temporal, bilateral | none | no | n.a. |
| 44 | DE | 0,41 | 3,18 | yes | temporo-occipital, r | 2 | 1 | (temporal, occipital), bilateral | FCD Ia | yes | 1A |
| 45 | DE | 8 | 37,34 | yes | fronto-parietal, l | 1 | 5 | (frontoal, parietal) l, frontal r | FCD IIb | yes | 1A |
| 46 | DE | 15 | 8,62 | yes | diffuse, bilateral | 3 | 2 | frontal, bilateral | none | no | n.a. |
| 47 | DE | 5 | 14,46 | yes | temporal, l | 0 | 3 | temporal | HS 1 | yes | 1A |
| 48 | DE | 10 | 17,54 | yes | temporal, l | 0 | 5 | temporal, parietal, frontal | HS 1 | yes | 1D |
| 49 | DE | 4 | 15,53 | yes | frontal, r | 5 | 0 | frontal, parietal | FCD IIb | yes | 1A |
| 50 | DE | 1 | 12,72 | yes | temporal, r | 7 | 0 | temporal, frontal, occipital | HS 1 + blurred grey-white-matter junction | yes | n.a. |
| 51 | DE | 4 | 40,93 | yes | diffuse, bilateral | 4 | 1 | (frontal, temporal) r, frontal l | none | no | n.a. |
| 52 | DE | 12 | 35,83 | yes | diffuse, bilateral | 3 | 3 | frontal, bilateral | none | no | n.a. |
| 53 | DE | 1 | 4,87 | yes | fronto-insular, l | 0 | 9 | frontal, temporal, parietal, insula | MOGHE | yes | 1A |
| 54 | DE | 1 | 48,82 | yes | temporo-occipital, r | 4 | 4 | (temporal, parietal) bilateral | none | no | n.a. |
| 55 | DE | 35 | 3,66 | yes | temporal, l | 3 | 3 | temporal, bilateral | HS | yes | 3A |
| 56 | DE | 1 | 9,29 | yes | frontal, r | 5 | 0 | frontal | FCD IIa | yes | 2A |
| 57 | DE | 3 | 11,65 | yes | frontal, l | 0 | 3 | frontal | none | yes | 1A |
| 58 | DE | 10 | 4,29 | yes | temporo-occipital, r | 3 | 0 | temporal, occipital | Ganglioglioma | yes | 1A |
| 59 | DE | 1 | 15,15 | yes | temporal, l | 0 | 6 | temporal, frontal, insula | HS 1 | yes | 1A |
| 60 | DE | 10 | 1,3 | no | diffuse, bilateral | 5 | 3 | frontal, bilateral | none | no | n.a. |
| 61 | DE | 4 | 21,83 | yes | diffuse, bilateral | 4 | 4 | (frontal, temporal), bilateral | none | no | n.a. |
| 62 | DE | 10 | 15,56 | no | temporal, r | 6 | 0 | frontal, temporal, parietal, insula | no pathology detected | yes | 1A |
| 63 | DE | 15 | 25,56 | yes | frontal, r | 8 | 0 | frontal, temporal, parietal, occipital | none | no | n.a. |
| 64 | DE | 11 | 15,18 | yes | temporal, l | 5 | 5 | (frontal, temporal), bilateral | HS 1 + FCD IIIa | yes | 1A |
| 65 | DE | 16 | 24,35 | no | temporal, r | 4 | 0 | frontal, temporal, parietal | mMCD II | yes | 2A |
| 66 | DE | 0,16 | 4,45 | no | hemispheric, l | 0 | 5 | frontal, parietal | blurred grey-white-matter junction (Hitsology from past operation) | no | n.a. |
| 67 | DE | 11 | 15,77 | no | frontal, l | 0 | 4 | frontal, parietal | FCD IIb | yes | 1B |
| 68 | DE | 1 | 17,53 | yes | temporal, l | 3 | 3 | temporal, bilateral | HS 1 | yes | 1D |
| 69 | DE | 1 | 14,77 | yes | diffuse (the heterotopia is not epileptogenic) | 3 | 0 | frontal | none | no | n.a. |
| 70 | DE | 3 | 3,44 | yes | fronto-temporo-insular, r | 7 | 0 | frontal, insula, temporal, parietal | MOGHE | yes | 1A |
| 71 | DE | 22 | 17,53 | yes | temporal, l | 3 | 3 | temporal, bilateral | HS 2 | yes | 1A |
| 72 | DE | 13 | 12,85 | yes | temporal, bilateral | 1 | 1 | temporal, bilateral | none | no | n.a. |
| 73 | DE | 15 | 19,35 | yes | temporal, bilateral | 3 | 1 | (temporo-occipital) r, temporal l | mMCD II | yes | 3A |
| 74 | DE | 1 | 25,61 | yes | temporo-occipital, l | 0 | 3 | temporal, occipital | mMCD II | yes | 4B |
| 75 | DE | 22 | 6,75 | yes | frontal, r | 4 | 0 | frontal | Gliosis | yes | 2B |
| 76 | DE | 17 | 11,52 | yes | temporal, l | 0 | 4 | temporal | HS | yes | 1A |
| 77 | DE | 3 | 6,87 | yes | frontal, l | 0 | 3 | frontal | none | yes | 1A |
| 78 | DE | 7 | 8,64 | yes | parieto-occipital, l | 0 | 2 | parietal, occipital | no pathology detected | yes | 4B |
