# Supplementary material for: Effect of stereo‐EEG versus subdural EEG on functional and seizure outcome in pediatric and adult epilepsy surgery: A 21‐year single‐center experience
Source: Epileptic Disord. 2025 May 13;27(4):586–99. doi: 10.1002/epd2.70025 (PMC12398195; doi:10.1002/epd2.70025)
Supplement: Supplementary file 2 — File S2. [file EPD2-27-586-s003.docx]

**Supplementary file with patient level data for subdural electrodes (SD):**

r: right, l: left, SOZ: seizure onset zone, HS: hippocampus sclerosis, FCD: focal cortical dysplasia, MOGHE: mild malformation of cortical development with oligondendroglial hyperplasia, mMCD: mild malformation of cortical development, DNET: dysembryonal neuroepithelial tumour, n.a.: not available.

| Patient Nr. | DE vs SD | Epilepsy onset | Epilepsy duration | MR positive | SOZ localization | number grids/strips right | number grids/strips left | electrodes localization | Histopathology | Surgery | Outcome Engel 2 years |
| --- | --- | --- | --- | --- | --- | --- | --- | --- | --- | --- | --- |
| 1 | SD | 1 | 53,6 | yes | temporal, l | 1 | 4 | (temporal, frontal) l, temporal r | HS 1 + blurred grey-white-matter junction | yes | 1A |
| 2 | SD | 25 | 4,1 | yes | temporal, l | 0 | 1 | temporal | Cavernous hemangioma | yes | 1A |
| 3 | SD | 25 | 22,6 | yes | temporal, r | 4 | 1 | (temporal, parietal, occipital) r, temporal l | HS1 + FCD IIIa | yes | 1A |
| 4 | SD | 31 | 7,3 | yes | temporal, l | 0 | 1 | temporal | Cavernous hemangioma | yes | 1A |
| 5 | SD | 15 | 9 | yes | temporal, l | 5 | 5 | (frontal, temporal), bilateral | HS 1 | yes | 3A |
| 6 | SD | 14 | 11,5 | yes | parieto-temporal, l | 0 | 1 | parietal, temporal | FCD IIb | yes | 1A |
| 7 | SD | 1 | 51,3 | yes | temporal, l | 0 | 4 | temporal | HS 1 + mMCD | yes | 1A |
| 8 | SD | 27 | 3,9 | no | temporal, r | 4 | 0 | temporal, parietal | Gliosis | yes | 1A |
| 9 | SD | 11 | 3,6 | yes | temporal, l | 0 | 1 | temporal | Gangliogliom | yes | 1A |
| 10 | SD | 22 | 13,2 | no | temporal, l | 4 | 4 | temporal, bilateral | blurred grey-white-matter junction | yes | 3A |
| 11 | SD | 99 | 99 | yes | temporal, l (extent not defined) | 0 | 1 | temporal, frontal, parietal | FCD IIb | yes | 1A |
| 12 | SD | 7 | 4,9 | yes | temporo-occipital, l | 0 | 4 | temporal, occipital | Gangliogliom | yes | 1A |
| 13 | SD | 2,3 | 2,3 | yes | frontal, l | 0 | 4 | frontal, parietal | MOGHE | yes | 2A |
| 14 | SD | 42 | 6,2 | no | temporal, r | 6 | 0 | temporal, frontal, parietal | blurred grey-white-matter junction | yes | 3A |
| 15 | SD | 50 | 2,4 | yes | frontal, l (extent not defined) | 0 | 2 | frontal, parietal | mMCD II | yes | 4B |
| 16 | SD | 7 | 15,4 | no | temporo-occipital, r | 8 | 0 | temporal, frontal, parietal, occipital | blurred grey-white-matter junction | yes | 1A |
| 17 | SD | 3 | 2,8 | yes | temporal, l | 0 | 1 | temporal | FCD IIb | yes | 1A |
| 18 | SD | 5 | 15,2 | yes | frontal, r | 1 | 0 | frontal, parietal | FCD IIb | yes | 1A |
| 19 | SD | 0,1 | 3,1 | yes | frontal, r | 1 | 0 | frontal, parietal | FCD IIb | yes | 1A |
| 20 | SD | 3 | 22,5 | yes | temporal, l | 0 | 4 | temporal, occipital | HS1 + blurred grey-white-matter junction | yes | 1A |
| 21 | SD | 3 | 31,2 | yes | temporal, l | 0 | 4 | temporal, occipital | HS1 + blurred grey-white-matter junction | yes | 4B |
| 22 | SD | 1,3 | 15,2 | yes | temporal, l | 0 | 4 | temporal, occipital | HS 1 + FCD IIb | yes | 1B |
| 23 | SD | 8 | 6 | yes | fronto-parietal, r | 2 | 0 | frontal, parietal | none | no | n.a. |
| 24 | SD | 8 | 35,7 | yes | frontal, l (extent not defined) | 1 | 2 | (frontal, parietal) l, frontal r | FCD IIb | yes | 4B |
| 25 | SD | 16 | 29,9 | yes | temporal, r | 4 | 1 | temporal, bilateral | mMCD II | yes | 3A |
| 26 | SD | 29 | 6 | yes | temporal, r | 4 | 0 | temporal | blurred grey-white-matter junction | yes | 1A |
| 27 | SD | 13 | 9,2 | yes | temporal, r | 5 | 1 | temporal, bilateral | HS 1 | yes | 4B |
| 28 | SD | 23 | 4,5 | no | temporal, l | 0 | 6 | temporal, occipital | mMCD II | yes | 1A |
| 29 | SD | 3 | 48,7 | yes | temporal, l | 0 | 5 | temporal | none | no | n.a. |
| 30 | SD | 13 | 21,4 | yes | temporo-parietal, l | 0 | 1 | temporal, parietal | Cavernous hemangioma | yes | 3A |
| 31 | SD | 14 | 13,7 | no | temporal, l | 0 | 4 | temporal | blurred grey-white-matter junction | yes | 4B |
| 32 | SD | 10 | 44,2 | yes | frontal, l | 2 | 2 | (temporal, occipital) r, (frontal, parietal) l | Cavernous hemangioma | yes | 2B |
| 33 | SD | 6 | 9,7 | yes | temporal, l | 0 | 5 | temporal | Angiozentrisch Glioma | yes | 1B |
| 34 | SD | 8 | 5,6 | yes | temporal, l | 0 | 1 | temporal, parietal | Complicated variant of a glio-neuronal Tumor | yes | 1B |
| 35 | SD | 5 | 6,4 | yes | temporal, l | 0 | 6 | temporal | Ganglioglioma | yes | 1A |
| 36 | SD | 99 | 99 | yes | temporal, r | 6 | 0 | temporal, frontal, occipital | mMCD II | yes | 3A |
| 37 | SD | 5 | 22,3 | no | fronto-parietal, r | 5 | 0 | frontal, parietal | FCD IIa | yes | 3A |
| 38 | SD | 14 | 13,5 | no | temporal, r | 5 | 0 | temporal, parietal | mMCD II | yes | n.a. |
| 39 | SD | 0,3 | 45,2 | yes | frontal, l | 0 | 2 | frontal | FCD IIa | yes | 1A |
| 40 | SD | 9 | 15,7 | yes | frontal, l | 0 | 1 | frontal, parietal | FCD IIa | yes | 1D |
| 41 | SD | 33 | 15,9 | no | temporal, r | 6 | 0 | temporal, occipital | blurred grey-white-matter junction | yes | 1A |
| 42 | SD | 2 | 7,4 | yes | temporo-occipital, l | 0 | 5 | temporal, occipital | HS 2 + Gangliglioma | yes | 2B |
| 43 | SD | 14 | 10,5 | yes | temporal, r | 7 | 1 | (frontal, parietal, temporal) r, temporal l | HS1 + blurred grey-white-matter junction | yes | 4B |
| 44 | SD | 36 | 8,2 | yes | temporal, r | 6 | 0 | frontal, parietal, temporal | no pathology detected | yes | 1A |
| 45 | SD | 10 | 20,8 | yes | diffuse bilateral | 4 | 2 | frontal, bilateral | none | no | n.a. |
| 46 | SD | 5 | 46,3 | yes | temporal, r | 6 | 0 | temporal, frontal, parietal | FCD IIb | yes | 2D |
| 47 | SD | 23 | 15,9 | no | temporal, r | 6 | 0 | temporal, frontal | FCD IIb | yes | 1A |
| 48 | SD | 5,5 | 7,2 | yes | temporo-occipital, l | 0 | 2 | temporal, occipital | Gliosis + FCD IIIb | yes | 1A |
| 49 | SD | 32 | 13,3 | no | temporal, l | 0 | 3 | temporal | mMCD II | yes | 2B |
| 50 | SD | 23 | 8,5 | no | temporal, r | 7 | 0 | temporal, frontal | HS 2 + FCD IIIa | yes | 1A |
| 51 | SD | 5 | 5,6 | yes | temporo-occipital, r | 5 | 0 | temporal, occipital | FCD Ia | yes | 1A |
| 52 | SD | 24 | 17,1 | yes | temporal, r | 2 | 2 | temporal, bilateral | HS 2 | yes | 1A |
| 53 | SD | 7,5 | 4,5 | yes | parietal, r | 1 | 0 | frontal, parietal | Gliosis + FCD IIId | yes | 3B |
| 54 | SD | 13 | 30,1 | yes | frontal, r | 1 | 0 | frontal, parietal | FCD IIb | yes | 4B |
| 55 | SD | 5 | 7,8 | yes | temporal, l (extent not defined) | 1 | 5 | (frontal, temporal) l, temporal r | mMCD II | yes | 1B |
| 56 | SD | 1,2 | 5,8 | yes | frontal, r | 1 | 0 | frontal, parietal | Cortical tuber | yes | 1A |
| 57 | SD | 14 | 14,3 | yes | temporal, l | 0 | 5 | frontal, temporal | HS1 + Gliosis | yes | 2B |
| 58 | SD | 0,5 | 2,9 | yes | fronto-temporal, l | 0 | 5 | frontal, temporal | Cortical tuber | yes | 4B |
| 59 | SD | 11 | 35,2 | yes | frontal, r | 5 | 0 | frontal, temporal | MOGHE | yes | 3A |
| 60 | SD | 0,9 | 8,2 | yes | temporo-occipital, l (extent not defined) | 0 | 4 | temporal, occipital | MOGHE | yes | 4B |
| 61 | SD | 0,3 | 14,5 | yes | temporo-parietal, l | 0 | 4 | temporal, parietal | Cortical tuber | yes | n.a. |
| 62 | SD | 18 | 24,6 | yes | temporal, r | 4 | 0 | temporal, occipital | HS | yes | 1A |
| 63 | SD | 17 | 3,6 | yes | frontal, r (extent not defined) | 3 | 0 | frontal | non-specific changes | yes | 2B |
| 64 | SD | 0,2 | 19,4 | yes | frontal, l | 3 | 3 | (frontal, temporal, parietal, occipital) bilateral | Cortical tuber | yes | 4B |
| 65 | SD | 6 | 24 | yes | temporal, l | 0 | 3 | temporal | HS + FCD IIa | yes | 2B |
| 66 | SD | 8 | 12,4 | yes | temporo-occipital, r | 1 | 2 | (temporal, occipital) bilateral | FCD IIIb + Ganglioglioma | yes | 1A |
| 67 | SD | 6 | 4,9 | yes | frontal, l | 0 | 2 | frontal | Cortical tuber | yes | 3A |
| 68 | SD | 6 | 30,9 | yes | frontal, l | 0 | 1 | frontal | Angiocentric neuroepithelial Tumor | yes | 4A |
| 69 | SD | 10 | 7,9 | yes | frontal, l | 0 | 1 | frontal | non specific changes | yes | 4B |
| 70 | SD | 3,5 | 17,2 | yes | frontal, r | 3 | 0 | frontal, parietal | FCD IIb | yes | 1A |
| 71 | SD | 2,8 | 9,1 | yes | temporal, l | 0 | 5 | frontal, temporal | Cortical tuber | yes | 1A |
| 72 | SD | 11 | 9,9 | yes | temporo-occipital, r | 3 | 0 | temporal, occipital | FCD Ic | yes | 1A |
| 73 | SD | 0,8 | 3,3 | yes | parietal, l | 0 | 1 | frontal, parietal | Cortical tuber | yes | 1A |
| 74 | SD | 12 | 19,7 | yes | frontal, r | 1 | 1 | frontal, bilateral | FCD Ib | yes | 3A |
| 75 | SD | 1 | 43,3 | yes | temporal, r | 2 | 2 | temporal, bilateral | HS | yes | 1A |
| 76 | SD | 3 | 34,8 | yes | frontal, l | 0 | 1 | frontal | FCD IIb | yes | 1B |
| 77 | SD | 3 | 28,8 | no | temporal, r | 5 | 0 | frontal, temporal | HS | yes | 4B |
| 78 | SD | 11 | 13,1 | yes | frontal, l | 0 | 1 | frontal, parietal | FCD IIb | yes | 4B |
| 79 | SD | 22 | 6,6 | no | temporal, l | 5 | 2 | temporal, bilateral | non specific changes | yes | 3A |
| 80 | SD | 0,1 | 1,6 | yes | frontal, l | 0 | 4 | frontal | mMCD II | yes | 4B |
| 81 | SD | 2,5 | 54,1 | yes | temporal, r | 3 | 2 | (temporal, parietal) r, temporal l | HS 1 + FCDIIb | yes | 1A |
| 82 | SD | 7 | 6,6 | yes | temporal, l (extent not defined due to bleeding and preterm explantation) | 0 | 7 | temporal, occipital, parietal | Ganglioglioma | yes | 1A |
| 83 | SD | 37 | 1,5 | yes | temporal, l | 0 | 1 | temporal | diffuse Glioma | yes | 1A |
| 84 | SD | 16 | 21,2 | yes | frontal, r | 1 | 0 | frontal, parietal | FCD IIb | yes | 1A |
| 85 | SD | 3 | 7,6 | yes | frontal, r | 4 | 0 | frontal | FCD IIb | yes | 1A |
| 86 | SD | 2,2 | 5,6 | yes | frontal, r (not well localized because of subural bleeding) | 5 | 0 | frontal | FCD IIb | yes | 4B |
| 87 | SD | 0,6 | 3,9 | yes | hemispheric, r | 2 | 0 | frontal, parietal | mMCD I | yes | 1D |
| 88 | SD | 14 | 8,1 | yes | temporal, l | 2 | 3 | temporal, bilateral | Ganglioglioma | yes | 3A |
| 89 | SD | 16 | 9,8 | yes | frontal, l | 0 | 5 | frontal | FCD IIa | yes | 3A |
| 90 | SD | 12 | 0,7 | yes | temporal, r | 2 | 0 | temporal | Ganglioglioma | yes | 1A |
| 91 | SD | 2 | 31 | yes | temporal, l | 0 | 4 | temporal, parietal, occipital | HS | yes | 1A |
| 92 | SD | 7 | 41,7 | yes | temporo-occipital, l | 2 | 4 | (temporal) bilateral, occipital l | HS | yes | 4B |
| 93 | SD | 13 | 11,5 | yes | frontal, r | 1 | 0 | frontal, parietal | non specific changes | yes | n.a. |
| 94 | SD | 5 | 23,4 | yes | temporal, l | 2 | 3 | temporal, bilateral | angiocentric Ganglioglioma | yes | 1A |
| 95 | SD | 0,8 | 47,8 | yes | temporal, l | 2 | 3 | temporal, bilateral | HS | yes | 1A |
| 96 | SD | 0,3 | 3 | yes | frontal, l (not well localized) | 0 | 3 | frontal | Cavernous hemangioma | yes | n.a. |
| 97 | SD | 2,4 | 6 | yes | frontal, r | 2 | 0 | frontal, parietal | MOGHE | yes | 1A |
| 98 | SD | 22 | 0,9 | yes | frontal, l | 0 | 1 | frontal | DNET | yes | 1A |
| 99 | SD | 2,8 | 22,4 | yes | temporal, l | 0 | 4 | temporal, frontal, parietal | HS + Gliosis | yes | 1A |
| 100 | SD | 4 | 50,2 | yes | temporal, r | 2 | 2 | temporal, bilateral | HS | yes | 3A |
| 101 | SD | 1,5 | 3,4 | yes | frontal, r | 3 | 0 | frontal, parietal, occipital | MOGHE | yes | 3A |
| 102 | SD | 23 | 24,2 | yes | temporal, l | 2 | 2 | temporal, bilateral | HS | yes | n.a. |
| 103 | SD | 21 | 5,4 | yes | temporo-occipital, l | 0 | 1 | temporal, occipital | Cavernous hemangioma | yes | n.a. |
| 104 | SD | 28 | 35,3 | yes | frontal, l | 0 | 1 | frontal | DNET | yes | 1A |
| 105 | SD | 0,6 | 4,4 | yes | frontal, l | 0 | 2 | frontal | Cortical tuber | yes | 1A |
| 106 | SD | 17 | 17,8 | yes | temporal, r | 2 | 2 | temporal, bilateral | HS | yes | 1A |
| 107 | SD | 5 | 37,1 | yes | frontal, l | 0 | 4 | frontal, temporal | FCD II | yes | 4B |
| 108 | SD | 5,5 | 9,7 | yes | temporal, l (not well localized due to brain odema and direct explantation) | 0 | 1 | temporal, parietal | Gliosis | yes | 4B |
| 109 | SD | 3 | 3,8 | yes | frontal, l (not well localized) | 0 | 3 | frontal, parietal, temporal | non specific changes | yes | 3A |
| 110 | SD | 14 | 19,8 | yes | temporo-occipital, l | 0 | 6 | temporal, parietal, occipital | FCD I | yes | 2B |
| 111 | SD | 7 | 4 | yes | fronto-parietal, l | 0 | 2 | frontal, parietal | DNET | yes | 1B |
| 112 | SD | 8 | 37,7 | yes | temporal, l | 1 | 2 | (temporal, frontal) l, temporal r | HS + FCD 1b | yes | 2B |
| 113 | SD | 8 | 37,5 | yes | temporal, l | 0 | 1 | temporal, frontal | DNET | yes | 1A |
| 114 | SD | 14 | 3 | yes | temporo-parietal, l | 0 | 2 | frontal, parietal, temporal | Ependymoma (biopsy, no epilepsy surgery) | no | n.a. |
| 115 | SD | 0,9 | 12,9 | yes | frontal, r | 3 | 0 | frontal, temporal | FCD IIa | yes | 1A |
| 116 | SD | 5,5 | 29,8 | yes | frontal, r | 2 | 0 | frontal | FCD IIb | yes | 1A |
| 117 | SD | 1 | 25,2 | yes | fronto-parietal, l | 0 | 1 | frontal, parietal | FCD IIb | yes | 1A |
| 118 | SD | 6 | 4,5 | yes | frontal, r | 1 | 0 | frontal | FCD IIa | yes | 1A |
| 119 | SD | 0,9 | 2,9 | yes | fronto-insular, r | 4 | 0 | frontal, temporal | FCD IIb | yes | 4B |
| 120 | SD | 10 | 5,3 | yes | frontal, r | 5 | 0 | frontal | FCD IIa | yes | 3A |
| 121 | SD | 12 | 5,9 | yes | parietal, l | 0 | 1 | frontal, parietal | arterio-vernous malformation + Gliosis | yes | n.a. |
| 122 | SD | 0,6 | 3,2 | yes | frontal, l | 0 | 4 | frontal | FCD IIb | yes | 1A |
| 123 | SD | 12 | 10,4 | yes | frontal, l | 0 | 2 | frontal | Gliosis | yes | 4B |
| 124 | SD | 1,8 | 4,7 | yes | frontal, l | 0 | 3 | frontal, parietal, temporal | FCD IIb | yes | 4B |
| 125 | SD | 3 | 7,3 | yes | frontal, l (extent not defined) | 0 | 3 | frontal | FCD I | yes | 2B |
| 126 | SD | 4 | 3,8 | yes | frontal, l | 0 | 2 | frontal | FCD II | yes | 1A |
| 127 | SD | 2 | 9,2 | yes | frontal, l | 0 | 4 | frontal | FCD I | yes | 4B |
| 128 | SD | 0,2 | 15,6 | yes | fronto-parietal, r | 1 | 0 | frontal, parietal | FCD I | yes | 4B |
| 129 | SD | 10 | 25,1 | yes | temporal, r | 3 | 0 | temporal, occipital | Ganglioglioma | yes | 1B |
| 130 | SD | 17 | 16,6 | yes | temporal, r | 1 | 1 | temporal, bilateral | non specific changes | yes | 2B |
| 131 | SD | 0,6 | 6,8 | yes | frontal, l | 0 | 2 | frontal, parietal | FCD II | yes | 4B |
| 132 | SD | 9 | 0,4 | yes | frontal, l | 0 | 2 | frontal | DNET | yes | 1A |
| 133 | SD | 2 | 7,1 | yes | fronto-temporal, r | 5 | 0 | frontal, temporal | FCD I | yes | 4B |
| 134 | SD | 12 | 19,4 | yes | temporal, l (extent not defined) | 0 | 2 | temporal | non specific changes | yes | 3A |
| 135 | SD | 10 | 10,9 | yes | fronto-temporal, r (extent not defined) | 3 | 0 | frontal, temporal | Gliosis | yes | 4B |
| 136 | SD | 17 | 13,9 | no | temporal, l | 1 | 1 | temporal, bilateral | none | no | n.a. |
| 137 | SD | 2 | 7,9 | no | frontal, l | 0 | 3 | frontal, temporal | FCD II | yes | 1A |
| 138 | SD | 13 | 3,9 | yes | temporal, l | 0 | 1 | temporal | DNET | yes | 1A |
| 139 | SD | 0,1 | 1,2 | yes | fronto-parieto-insular, l | 0 | 3 | frontal, parietal, occipital | FCD IIb | yes | 3A |
| 140 | SD | 3 | 8,9 | yes | frontal, r | 4 | 0 | frontal, temporal | FCD I | yes | 4B |
| 141 | SD | 7 | 5,5 | yes | temporo-occipital, l | 0 | 4 | temporal, occipital | FCD I + Gliosis | yes | 1A |
