# Supplementary material for: Effect of stereo‐EEG versus subdural EEG on functional and seizure outcome in pediatric and adult epilepsy surgery: A 21‐year single‐center experience
Source: Epileptic Disord. 2025 May 13;27(4):586–99. doi: 10.1002/epd2.70025 (PMC12398195; doi:10.1002/epd2.70025)
Supplement: Supplementary file 3 — Data S1. [file EPD2-27-586-s001.docx]

Answers

1. A

2. B

3. C
